# The Future of Islet Transplantation Is Now

**DOI:** 10.3389/fmed.2018.00202

**Published:** 2018-07-13

**Authors:** Rita Bottino, Michael F. Knoll, Carmela A. Knoll, Suzanne Bertera, Massimo M. Trucco

**Affiliations:** ^1^Institute of Cellular Therapeutics, Allegheny Health Network Research Institute, Allegheny Health Network, Pittsburgh, PA, United States; ^2^Department of Biological Sciences, Carnegie Mellon University, Pittsburgh, PA, United States; ^3^College of Medicine, Drexel University, Philadelphia, PA, United States

**Keywords:** islets, allotransplantation, type 1 diabetes, transplantation, pancreas, clinical islet transplantation, hypoglycemia, insulin independence

## Abstract

Milestones in the history of diabetes therapy include the discovery of insulin and successful methods of beta cell replacement including whole pancreas and islet cell transplantation options. While pancreas transplantation remains the gold standard for patients who have difficulty controlling their symptoms with exogenous insulin, islet allotransplantation is now able to provide similar results with some advantages that make it an attractive potential alternative. The Edmonton Protocol, which incorporated a large dose of islets from multiple donors with steroid-free immunosuppression helped to establish the modern era of islet transplantation almost 20 years ago. While islet allotransplantation is recognized around the world as a powerful clinical therapy for type 1 diabetes it is not yet recognized by the Federal Drug Administration of the United States. Large-scale clinical trials administered by the Clinical Islet Transplantation Consortium have recently demonstrated that the well-regulated manufacture of a human islet product transplanted into patients with difficult to control type 1 diabetes and with a history of severe hyperglycemic episodes can safely and efficaciously maintain glycemic balance and eliminate the most severe complications associated with diabetes. The results of these clinical trials have established a strong basis for licensure of clinical islet allotransplantation in the US. Recognition by the Federal Drug Administration would likely lead to third party reimbursement for islet allotransplantation as a therapeutic option in the United States and would make the treatment available to many more patients. The high costs of rampant diabetes justify the expense of the treatment, which is in-line with the costs of clinical pancreas transplantation. While much enthusiasm and hope is raised toward the development and optimization of stem cell therapy, the islet transplantation community should push toward licensure, if that means broader access of this procedure to patients who may benefit from it. Even as we prepare to take the first steps in that direction, we must acknowledge the new challenges that a shift from the experimental to clinical will bring. Clinical islet allotransplantation in the United States would be a game-changing event in the treatment of type 1 diabetes and also generate enthusiasm for continued research.

## Introduction

The central roles of the pancreas and insulin producing islets of Langerhans in the development of diabetes were established in the late nineteenth century. For over 50 years, islet transplantation has been rigorously studied in animal models with the hope of establishing a basis for human transplantation. After the recently concluded and successful phase 3 clinical trials conducted by the Clinical Islet Transplantation Consortium, we have the best chance yet to establish islet allotransplantation as a treatment for type 1 diabetes in the US, as it is already recognized in other parts of the world. If the Federal Drug Administration (FDA) were to finally license islet allotransplantation, major US insurance companies would most likely follow suit to establish the procedure as a covered treatment, making it available to many more individuals who could benefit from the therapy. It is important that the islet transplantation community pursues FDA licensure to bring islet therapy to the clinic before newer treatments options can overtake it.

## The history of diabetes before the discovery of insulin

Physicians from antiquity knew of a disease that caused excessive output of urine that had sweetness to it, that today we would call diabetes mellitus. Medical literature from the great centers of learning in the ancient world including Egypt, China, and India documented early attempts to categorize this disease (1, 2). By 150 AD, the Greek physician Arateus was able to provide a recognizable diagnosis and coined the name diabetes (meaning siphon) (1–3). Without a better understanding of the disease, which was well beyond their means, ancient physicians could do little more than prescribe dietary changes and watch their patients die. Gradually, as the practice of medicine became more of a science, a better understanding of the disease and its causes came into focus. While the honey-like sweetness (*mel, mellis*) of urine had long been associated with diabetes, [mellitus being added to the name in late seventeenth century (1, 2, 4), it was not until 1776 that Matthew Dobson was able to scientifically demonstrate excess sugar in the urine of those effected by the disease in his famous series of experiments, by boiling urine to dryness and observing the crystalized remains (1, 2, 4). In 1850 Claude Bernard discovered a substance in the liver that he named glycogen, which was converted into glucose and then secreted into the blood, an action that he believed to be the cause of diabetes (1, 2, 4). The second half of the nineteenth century saw scientific discussions centered on whether the primary cause of diabetes rested in the kidney or the pancreas. German physiologist Paul Langerhans first described the cells that would eventually be known as islets of Langerhans in 1869, although it would be left to others, years later, to determine their purpose (2, 5). In 1882, Oskar Minkowski and Joseph von Mering definitively concluded that diabetes was a disease of the pancreas by removing the pancreas from a dog, leading to diabetes. Transplantation of autologous pancreatic fragments under the skin showed transient improvement in glycosuria (1, 2, 6). Many experiments were conducted even into the twentieth century in which pancreatic fragments were transplanted into animals in mostly unsuccessful attempts to cure diabetes (7, 8).

## Islet transplantation in the twentieth century

Beginning with the discovery of insulin by Frederick Banting and Charles Best in 1922 (1, 2, 9), for which Banting, but not Best, won a Nobel Prize the following year, diabetes went from being a terminal disease to a chronic one. Refinements in the understanding of the human insulin sequence eventually led to the production of recombinant human insulin which replaced animal extracted insulin (10). While the multiple failures of pancreatic fragment transplantation and, more importantly, the beginning of insulin therapy curtailed further experimentation with pancreatic fragments, ideas destined to play significant roles in treatment of diabetes thus transplantation of the tissues that produce insulin were first broached in this era. One hypothesis first elucidated at this time was that exocrine acinar tissue was detrimental to the viability and function of the endocrine pancreatic graft. This led to the separation of endocrine tissue from the exocrine tissue before transplantation, which was first proposed in 1902 and finally implemented in the 1960s (6). Early methods of separation described by Claes Hellerstroem required arduous microdissection under the microscope (11) and, perhaps not surprisingly, due to the small amount of endocrine tissue available, did not immediately reinvigorate diabetes research efforts focused on animal pancreatic fragment transplantation. It was not until the discovery that collagenases broke down pancreatic fragments and the introduction of collagenase-based enzymatic processing described by Stanislaw Moskalewski in 1965 that research took off again (12). The renewed enthusiasm and research efforts of many lead to Paul Lacy's dual phase technique of islet dissociation and purification which became the standard for rodent islet isolation and led to an explosion of studies on pancreatic islets in rodents (13). In 1972 Ballinger and Lacy achieved the first experimental reversal of diabetes in rats using transplanted islets (14). Soon afterwards, Kemp et al demonstrated that islets infused via portal vein to the liver of rats was superior to intraperitoneal infusion (15), thus establishing the site of choice for clinical islet infusion that remains primary to this day.

The results of these studies led to a sustained burst of research utilizing rodent models, which were invaluable to the advancement of islet isolation, yet the techniques thus established were unsuccessfully in large animal studies. This lead to the removal of the purification steps in an attempt to maintain sufficient tissue to reverse diabetes in large animals. By the late 1970s, Horaguchi and Merrell developed a novel technique, which allowed the recovery of sufficient islets for transplantation in dogs. Their procedure consisted of intraductal injection of collagenase, mechanical dissociation of islets and digestion at 37°C, and filtrations through a 400 UM filter mesh (16). This, in turn, allowed Ray Rajotte and colleagues at the University of Alberta to develop advanced experimental models of immunosuppression and islet cryopreservation (17). By the end of the 1970s doctors at the University of Minnesota began applying the techniques developed in the laboratory over the last decade for clinical application in patients with type 1 diabetes (T1D). Despite resulting in poor metabolic control and the inability to solve crucial problems associated with inadequate immunosuppression, this proved to be a valuable step toward eventual success (18, 19). Around this same time, and with better results, some of the same physicians at the University of Minnesota carried out the first autologous islet transplantations as part of a palliative therapy in patients suffering from chronic pancreatitis and undergoing total pancreatectomy (20).

In 1979, a group from Zurich University led by Largiadèr, Kolb, and Binswanger reported the first successful transplantation of allogeneic pancreatic fragments in conjunction with a kidney transplant in a patient with T1D, culminating in 10 months of exogenous insulin independence until kidney failure (21, 22). The University of Miami reported promising results of allogeneic islet transplantation in 1985 although ultimately ending in graft failure, which was determined to be most likely due to inadequate immunosuppression (6). A major turning point in islet transplantation occurred in 1988 when Camillo Ricordi, working with Lacy at Washington University in St. Louis, developed his automated method of pancreas dissociation. This method consists of a chamber that allows mechanically enhanced enzymatic digestion in a dissociation/filtration system in which dissociated islets exit to avoid over-digestion (6, 23). The development of the Ricordi automated system allowed clinical islet transplantation to begin in earnest and it remains, to this day, one of the bedrocks of human and large animal islet isolation.

During the 1980s and 90s several groups continued to improve the techniques of islet isolation and the outcomes of islet transplantation resulting in many new protocols being developed throughout the world. Semiautomated density gradient separation of islets was introduced by Stephen Lake at the Leicester Royal Infirmary (24), while doctors at the University of Miami and Washington University were among those who improved gradients and added cold preservation solutions to enhance successful outcomes (25, 26).

A team at the University of Pittsburgh under the direction of Thomas Starzl and Camillo Ricordi confirmed the ability of allogeneic islet transplantation to restore long-term normoglycemia in immunosuppressed diabetic (but not T1D) patients. They reported on 9 patients who became diabetic after upper-abdominal exenteration and liver transplantation to remove extensive tumors. Patients received islets from either the liver donor or from a pool including an additional donor, with most maintaining sustained insulin independence or near independence for over 6 months. These cases, however, were without the added complications of T1D autoimmunity (27).

By 1990 insulin independence in T1D patients following intraportal islet transplantation utilizing a single islet source was reported at the San Raffaele Institute, Milan, Italy (28) and, utilizing a pool of two islet preparations (of which some islets were cryopreserved), at Washington University (29).

Continued trials in Pittsburgh and Miami incorporated gravity infusion of the islets to reduce portal pressure and reduce the risk of portal hypertension (30). The trials demonstrated long-term (>16 mo) insulin independence but concluded that standard steroid based immunosuppression was problematic for engraftment (30).

The Islet Transplant Registry collected data from 267 islet transplants voluntarily reported by several Centers from 1990 to 2001. It reported that 12.4% of cases achieved insulin independence for periods of at least 1 week, with 8.2% reporting periods greater than 1 year (6). The results were promising but not consistent. Proper immunosuppression remained a key piece of the puzzle yet to be figured out in order to improve long-term graft function and consistent insulin independence.

## A new millennium begins

The year 2000 was a milestone in the advancement of islet transplantation with the publication of the Edmonton Protocol, which produced results far more promising than what had been previously reported in the registry. Seven consecutive patients with T1D that was difficult to control using standard therapies reported 100% insulin independence at 1 year. Novel features of the protocol included the transplantation of a large number of fresh islets from more than one donor, without culture, human albumin instead of fetal bovine serum in cell-processing medium, and, importantly, steroid-free immunosuppression based on sirolimus, tacrolimus, and anti-IL-2 antibody (31). It seemed, at last, that the obstacle presented by immunosuppression had finally been overcome. The future was promising for islet allotransplantation to become a primary therapeutic option for T1D.

Other centers were quick to attempt to duplicate the success of Edmonton. A subsequent multi-center international trial of the Edmonton Protocol achieved 58% insulin independence at 1 year, still a significant jump from previous efforts, yet also highlighting the importance of hard-won experience in islet processing and patient management that was not yet well established at many centers (32, 33). The Edmonton Protocol was then widely adopted either intact or in a modified form by many centers worldwide.

The extended follow-up from these trials demonstrated progressive loss of insulin independence over time and the need to renew exogenous insulin. While up to 80% of patients showed sustained graft function as defined by the presence of C-peptide, only 10% demonstrated independence from exogenous insulin at 5 years after transplantation (34). Even without insulin independence, however, some benefits of islet transplantation were observed such as the stabilization of glycemic control that would later take on more importance in discussions of the risk to reward ratio for the procedure. While islet allotransplantation remained an interesting experimental treatment, the road to the clinic remained longer than hoped.

## After the edmonton protocol

After the initial success of the Edmonton Protocol and the positive results of the international multi-center trial, the islet transplantation community began to greatly expand. In 2001, the Collaborative Islet Transplant Registry (CITR) was established to collect and share data among members. Over 30 centers from around the world voluntarily reported their activities to CITR while an undetermined number choose not to participate (6).

While the establishment of the Edmonton Protocol was immediately looked upon as the beginning of an epoch in islet transplantation, several advancements that were developed later have contributed significantly to the current state of islet transplantation. The adoption of new pancreas preservation techniques has enhanced the effectiveness of University of Wisconsin (UW) Solution for pancreas transportation (35–37) leading to higher islet yields. The description of instant blood mediated inflammatory response (IBMIR) after the portal infusion of islets by Olle Korsgren and colleagues at the Uppsala University and Karolinska Institute in Sweden (38) led to targeted anti-inflammatory strategies aimed at improving graft survival. Doctors at the University of Miami and other centers incorporated tumor necrosis factor (TNF) alpha-blockers to supplemental islet infusions (39), where they have been associated with improved results. This has been further evidenced with the combination of TNF-alpha and interleukin (IL)-1-beta blockers at the Baylor Research Institute (40).

## The clinical islet transplant consortium

A major development in the field of islet transplantation after the success of the Edmonton Protocol was the combination of individual centers into larger groups such as the GRAGIL network in France and Switzerland, the Nordic Network for Clinical Islet Transplantation (NNCIT) in the Scandinavian countries and the Clinical Islet Transplant Consortium (CITC) internationally but concentrated in North America. These consortia allowed larger groups to take advantage of the experience earned at top centers, facilitated the sharing of information between members, and helped streamline procurement, islet isolation, and transplantation. Perhaps the most significant grouping, the CITC was established by the National Institutes of Health (NIH) in 2004 and sponsored by the National Institute of Diabetes and Digestive and Kidney Diseases (NIDDK) and the National Institute of Allergy and Infectious Diseases (NIAID). Its multifaceted mission was to conduct studies aimed at improving the isolation and viability of islets, reducing complications associated with the transplant procedure and side effects of immunosuppression, to achieve good blood glucose control without hypoglycemia, to determine what happens to islets after transplantation, why they sometimes fail, and to evaluate new methods to prevent immune rejection (41). The CITC has brought together many of the renowned centers for islet transplantation to become, in effect, “the face” and the engine driving the islet transplantation community. To complete its mission, the CITC designed, standardized, and optimized protocols to regulate large-scale multi-center international clinical trials of islet allotransplantation.

**Figure 1:**
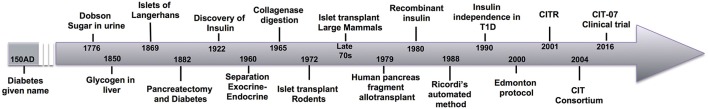
A historical timeline of significant events in the progression of scientific investigation culminating in the successful clinical trials of islet allotransplantation.

For a timeline of the discussed events, see Figure 1.

The Clinical Islet Transplantation (CIT)-06 protocol regulated a phase 3 clinical trial on the efficacy of islet after kidney transplantation. Subjects who had previously been diagnosed with End Stage Renal Disease (ESRD) and had undergone kidney transplantation, who were diagnosed with T1D, and satisfied all other eligibility criteria were recruited into this multi-center study to undergo islet transplantation. Subjects received up to 3 separate islet transplantations. Immunosuppression required to prevent rejection of the kidney transplant was maintained with the addition of an induction protocol consisting of antithymocyte globulin (ATG), with Daclizumab or Basiliximab (IL-2 receptor antagonists) replacing ATG for subsequent transplantations and etanercept (Anti TNFalpha antibodies) at the time of islet allotransplantation.

Islet transplantation has always been an easier sell when the recipient is already committed to life-long immunosuppression (for example, from kidney transplant), thus, removing one of the major stumbling blocks for it's utilization.

Subjects who were diagnosed with T1D but who had not undergone kidney transplantation (not diagnosed with ESRD) were randomly assigned to either a phase 3 multi-center clinical trial of islet transplantation regulated under CIT-07 protocols or site specific clinical studies investigating specific immunosuppressive agents [CIT-02, lisofylline, CIT-03, deoxyspergualin, CIT-04, LEA29Y (Belatacept), CIT-0501, rituximab] in the context of islet transplantation only.

Under CIT-07, subjects received up to 3 separate islet transplantations from carefully chosen donors (although the majority received 1 or 2). The immunosuppressive regimen consisted of ATG, sirolimus (Rapamycin), and tacrolimus (FK-506). Basiliximab was used in place of ATG after the initial transplantation in subjects who required multiple doses of islets. Subjects also received a regimen of etanercept.

CIT-08 was an observational trial that provided extended follow-up for 75 subjects who had completed one of the parent CIT studies in order to collect islet function data and information about the safety of the medications that were required by the CIT protocols.

The islet transplant community eagerly awaited the results of these clinical trials. In 2016, Bernard Hering *et al* reported on the outcome of CIT-07, more specifically on the safety and effectiveness of a purified human pancreatic islet (PHPI) product transplanted to T1D subjects with impaired awareness of hypoglycemia (IAH) and severe hypoglycemic events (SHEs) (42). The primary endpoint was the measurement of HbA1c <7.0% at 1 year after initial transplantation with no reported SHEs after day 28. These conditions were achieved by 87.5% of subjects while 71% met them at 2 years. As a secondary measurement, insulin independence was reported as 52.1% at 1 year, falling to 42% at 2 years. Several subjects who either withdrew their consent for the study or were not able to be evaluated at the time point were included in data as failures and potentially skewed the overall results lower. More importantly, recipients also demonstrated significant improvements in additional measures of glycemic control and the restoration of hypoglycemia awareness even without insulin independence.

In year 1, 22 serious adverse events (SAEs) were attributed to the transplant procedure or immunosuppression and 2 more in year 2. Immunosuppression was responsible for 43% of SAEs and included a known and expected diminution of renal function associated with drugs used in the study.

The clinical study regulated under CIT-07 was the first phase 3 trial of any therapy to demonstrate effectively restoration of sustained normoglycemia while offering protection from SHEs for patients with long-standing T1D and a history of SHEs. Hering concluded that in subjects with IAH and SHEs, islet allotransplantation provides glycemic control, restores hypoglycemia awareness, and protects the recipient from SHEs. However, until further development of effective and less harmful immunosuppression, it was recommended only for the 20–30% of patients with T1D who suffer from SHEs despite exhausting all less invasive approaches of treatment (42).

**Figure 2:**
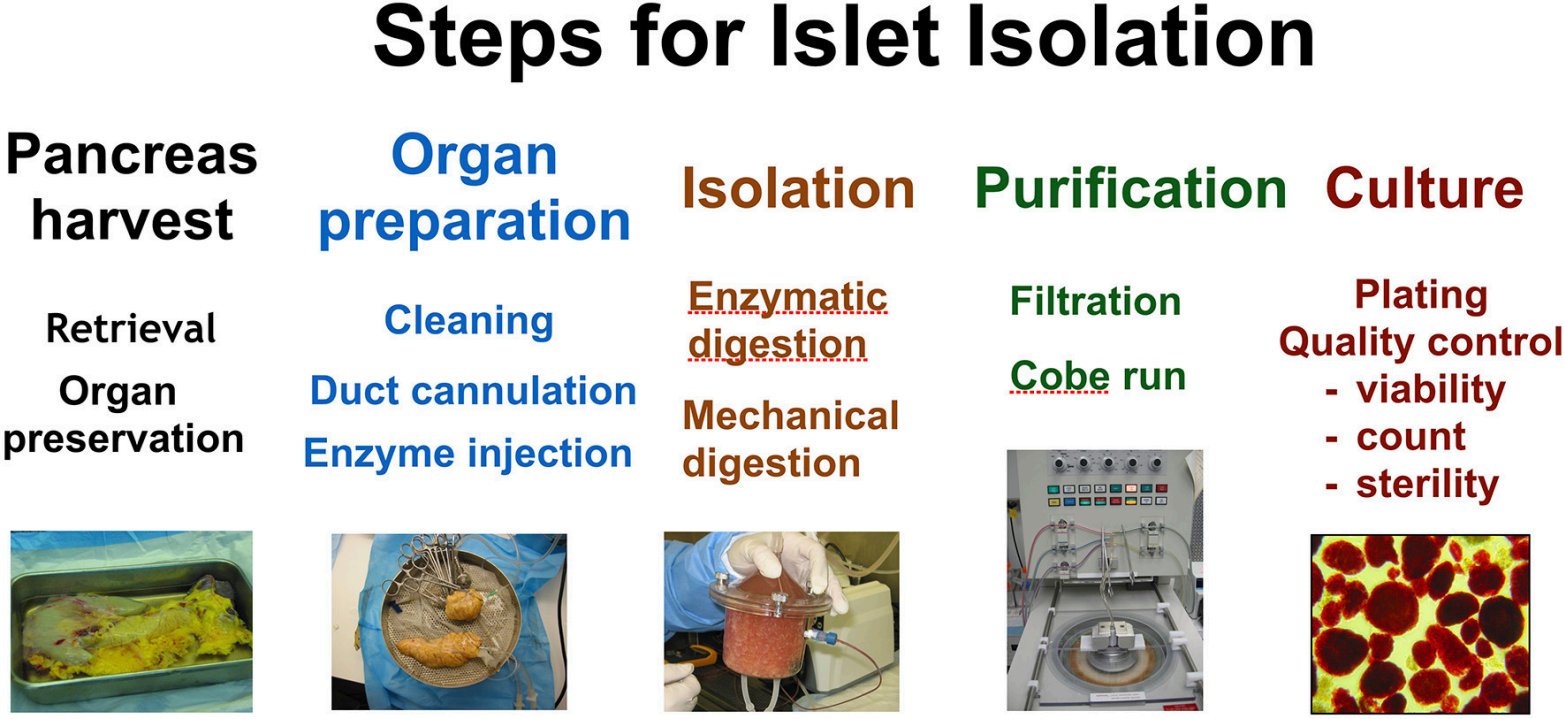
The major steps undertaken in islet isolation. From left to right; pancreas harvest, organ preparation, isolation, purification, and culture.

Later that year, Ricordi et al reported on the manufacturing process required to produce PHPI for allotransplantation under protocol CIT-07 (43). It is well known that the quality of islets for transplantation is affected by characteristics of the donor and the organ recovery process; therefore, in order to provide a consistent and successful product for transplantation, the CITC established stringent selection criteria and optimal organ recovery practices. PHPI were produced using Current Good Manufacturing Practices and Current Good Tissue Practices (see Figure 2).Just over 50% of the lots of PHPI prepared by all centers met the established release criteria. At individual centers, the success rate of PHPI released for transplantation ranged from 24 to 90% of the total number of islet lots manufactured.

Pancreata were accepted from donors between the age of 15–65 years, with a maximum of 12 h of cold ischemia, transported in an acceptable UW, perfluorinated compound (PFC)/UW, histidine-tryptofane-ketoglutarate (HTK), or PFC/HTK preservation solution, with cause and circumstances of death acceptable to the transplantation team. Donors were 71% male with median age of 42 years. Cause of death was 45% head trauma, 44% cerebrovascular accident, 5% anoxia. 9% of donors were known to undergo cardiac arrest, with 12% unknown. Islet equivalent quotient (IEQ) correlated with donor gender and body mass index (BMI) but no other donor or organ characteristics. The results of the clinical trial demonstrated that the PHPI products were safe, well tolerated, and effective in the specific subset of T1D population in which they were tested. The manufacturing processes yielded products that met the pre-specified criteria for safety, purity, potency, and identity (43). Additionally, patient surveys and qualitative analysis demonstrated significant improvement in quality of life (QOL) for the patients who underwent islet transplantation in the CITC clinical trials (44). Data collected from this trial will be made available to facilitate FDA licensure of PHPI as a regulated product, which, it is hoped, will increase patient access to islet transplantation along with third-party insurance coverage. Given previous differences in protocol implementation that contributed to widely divergent outcomes and patient experiences after islet transplantation, the task accomplished by the CITC has been herculean.

## The technical challenge of islet purification

The CIT-07 clinical trials were rigorous studies designed to demonstrate that islet allotransplantation as a therapeutic option for T1D patients had come of age, but they left room for several questions. The use of islet purification and the current methods of achieving the recommended purity are perhaps the most relevant area for further study. Islet purification is a central tenant of both the Edmonton protocol and, even more rigidly so, the CIT protocols, considered necessary in order to keep the islet product volume at manageable levels for intraportal infusion without increasing the risk of portal vein thrombosis (PVT). Even under current standards, PVT can be a serious even fatal risk in a small number of patients. Because of this, clinical islet transplantation is limited to 15 mL volume of tissue to be infused (43). Since CIT-07 requires a minimum of 5,000 IEQ/kg of subject body weight in order for a lot to be considered for transplantation, purification is necessary. By maximizing the insulin secreting beta cells in a limited volume to be transplanted the likelihood of a successful outcome is increased. The current gold standard and the method codified by CIT-07, is a density gradient centrifugation using a COBE 2991 cell processor. According to CIT-07 protocols, islet purity must be >20,000 IEQ/mL of settled tissue volume to be considered for transplantation and lots that are too impure or do not contain enough islets for transplantation are discarded unused (43).

One unavoidable drawback to purification, however, is that the centrifugation process destroys many of the cells, reducing yield, often below the needed threshold required for transplantation according to protocol. It was reported in the CIT-07 clinical trial that 18% of islets on average were lost after purification (43). The CITC also reported that, overall 47.5% of islet lots were not classified as PHPI to be used for transplantation in their clinical trials, with the most common reason being insufficient islet yield (43). It has been reported that in some experienced groups, up to 50% of clinically isolated islets are not used for transplantation because the required number of islets to transplant is not available after purification (45). This leads to a tremendous waste of valuable islets and adds unnecessary expense to an already expensive procedure.

Potentially, islets that do not meet the required level of purity could still provide the same benefits to patients as a more purified product. Alternative methods of islet purification are currently being explored. Ichii and colleagues at the University of Miami have suggested that rescue purification, or a re-purification of a highly mantled fraction, can often provide enough additional functional islets to bring the post-purification product back above the threshold for transplantation (46).

Autologous islet transplantation, in which non- purified islets can be transplanted back into a patient after pancreatectomy, demonstrates success in transplanting more impure islets while also showing insulin independence longer lasting than in islet allotransplantation. While this may be attributed to differences between allo- and autotransplantation, it at least demonstrates that highly impure islets are well tolerated in humans when precautions are taken to prevent rising portal pressure (47). Interestingly, even before the Edmonton Protocol, the University of Minnesota reported up to 6 months of insulin independence (a significant period of time for that era) after the allotransplantation of a very large amount of impure islets (48). Intrigued by this result, they then demonstrated the superiority of islet function after transplantation of less pure islets when compared to more pure islets in canines. After transplantation into the peritoneal cavity of canines <50% of purified islets remained functioning 3 weeks after transplantation while 67% of non-purified islets remained functional 6 months after transplantation (49).

One possible reason for less favorable outcomes for purified islet transplantation is that the purification step eliminates mantled islets, those that are surrounded by an extra cellular matrix (ECM) or acinar cells. Mantled islets are collected into a different density gradient fraction than purer islets during centrifugation, and generally discarded as impure “junk” as part of the allo-islet procedure leaving fewer islets to potentially transplant.

ECM provides structure to the islets and, thru complex interplay and communication, might help them to survive (50). The isolation process already damages the relationship between ECM and islets. Purification through current methods tends to remove islets surrounded by ECM. Thus, the mantled islets, which may promote islet survival in their new microenvironment, are selectively targeted for elimination, leaving the islets “helpless” in their new home. Older and obese donors are specifically preferred for clinical islet transplantation in a large part due to the ease in which the intact islets can be released from the surrounding acinar tissue and purified (51). Islets from younger donors are associated with superior function (52), however, young donors also tend to produce more mantled islets, making them less likely candidates to provide a large yield of highly pure islets using standard purification methods. Conventional wisdom focuses islet isolation efforts on the donors most likely to provide a successful isolation, generally defined by the same criteria as established by the CITC, thus potential donors that may provide perfectly capable islets are routinely ignored under current recommendations. Given the importance of the ECM, it is likely that selective donation in this regard has had some negative impact on the success of clinical engraftment by removing too much of the supporting tissue. Likewise, purification removes ductal epithelial cells, which have demonstrated angiogenic potential that may lead to increased islet vascularization (53), which is critical to graft survival.

The vast majority of literature on the subject is in favor of only highly purified islets being transplanted in order to reduce the risk of PVT, however, if purification is to remain an essential part of the islet isolation process, better methods must be developed to maximize islet yield and increase the chance of long-term islet function.

## What comes next?

In retrospect it seems obvious that a large consortium such as the CITC consisting of many of the top centers and experts in islet transplantation would be necessary to convince the FDA of the feasibility, safety and effectiveness of clinical islet allotransplantation. Currently Australia, several provinces of Canada, France, Italy, Switzerland, and the UK already consider islet transplantation for the treatment of T1D a reimbursable expense. In the US, islet autotransplantation in the context of total pancreatectomy for chronic pancreatitis is considered reimbursable but not islet allotransplantation in individuals diagnosed with T1D. Whole organ pancreatic transplantation is the only clinical beta cell replacement method that is reimbursed by third party insurance in the US for a diagnosis of T1D.

Four elements of the CIT clinical trials are key to potential FDA licensure, (1): replacing the outdated insulin independence standard of success with a more clinically relevant target, (2): consistently demonstrating success at reaching the clinically relevant target, (3): the real and significant relief experienced by subjects who receive islet therapy, and (4): the establishment of universal protocols to make an identifiable, reproducible, safe, effective islet product for transplantation.

There is a strong hope that FDA approval would lead to third party reimbursement for islet transplantation. Even under CIT recommendations limiting the procedure to patients with IAH and experiencing SHEs, recognition by the FDA and insurance companies would allow far more patients access to islet transplantation than have been able to take advantage of experimental trials.

While earlier attempts at islet transplantation could not match the long-term graft function provided by pancreas transplantation, the gap is narrowing, especially in experienced centers following CIT-07 protocols. Glycemic control after islet transplantation has been demonstrated to be comparable to that following whole pancreas (54, 55). In addition to providing glycemic control, islet allotransplantation therapy provides benefits toward reducing or eliminating diabetic complications similar to the effect found in pancreas transplantation but not observed with the daily intake of exogenous insulin. Diabetes can lead to secondary ocular, neurological, cardiovascular, and renal impairments as reviewed by Bassi et al. These complications can range from debilitating to life threatening. Islet transplantation has demonstrated to stabilize retinopathy, microangiopathy, polyneuropathy, improve diastolic function and improve kidney graft function (56). While the main goal of islet transplantation is to re-establish glycemic control and eliminate SHEs, transplanted islets producing endogenous insulin also has a positive impact on diabetic complications and consequent improvements in quality of life (57).

Due to the unsuitability of some patients for whole pancreas transplant and the unsuitability of some donors for pancreas transplantation, whole pancreas and islet cell replacement therapies may provide complementary therapeutic options for patients rather than compete for pancreas donors (58). Regardless, we may soon arrive at a point when islet allotransplantation may be the preferred method of beta cell replacement. A comparative cost analysis of islet versus pancreas transplantation in a single US Center reported in 2016 placed the cost for islet transplantation, including an average post-transplantation hospital stay of 5.75 days, at $138,872. The cost for pancreas transplantation was estimated to be $134,748, including an average post-transplantation hospital stay of 12 days. The majority of patients who underwent pancreas transplantation, but none that underwent islet transplantation, reported complications that required further in-patient care over 4 years of follow-up. Even more remarkable, the calculated cost of islet transplantation factored in a second islet infusion needed by almost half of the recipients, which contributed to the higher average cost of the procedure (54). As CIT-07 demonstrates, under the most current methods, subjects undergoing islet transplantation more often require only a single transplant (55, 58). The trend should continue in this direction, reducing the cost of islets even more. The cost for a single donor islet procedure was estimated to be <$100,000, representing a significant savings over pancreas transplantation (54).

Islet allotransplantation is a less invasive procedure than pancreas transplantation and evidence indicates that it presents patients with fewer risks of serious complications while producing similarly effective outcomes. When all of these factors are considered, it would seem an appropriate time for insurance companies in those countries where islet transplantation is yet a non-reimbursable procedure for T1D to rethink their position on islet allotransplantation.

The high cost associated with islet therapy should not be a primary consideration in the decision-making process to consider third party reimbursement given the similar cost of the already accepted pancreas transplantation. This is especially true in light of the ever-increasing financial burden that diabetes places on society, a burden that islet transplantation can help to relieve. A calculation of the economic cost of diabetes in the US, published by the American Diabetes Association in 2017, places the cost of diagnosed diabetes at $237 billion in direct care with an additional $90 billion in lost productivity. Costs of treatment have risen by 26% over the last 5 years (adjusted for inflation), a trend likely to continue as the population ages. Individuals diagnosed with diabetes have 2.3 X higher yearly medical expenditures than they would in the absence of diabetes, almost $17,000 per year (59). A team at the University of Minnesota recently calculated that, even factoring in multiple infusions, islet transplantation is more cost effective than standard insulin based therapy within 10 years after the start of therapy and is more effective immediately (60). These significant monetary figures do not even begin to take into account the additional years of life and QOL associated with overcoming diabetes and its complications, which can be priceless and extend beyond the individual to families and communities.

## Extending the reach of islet transplantation

The credibility provided by FDA approval would likely spur interest in continued research and facilitate the next generation of islet products that will provide a larger pool of islets for transplantation, thus allowing islet therapy to expand to include even more of the T1D population. New sources of islets may include islets from marginal donors outside of currently recommended donor characteristics, xenogeneic islets, or encapsulated islets, or even stem cell derived islets.

Methods for optimizing islet recovery from marginal donors have been pioneered by several centers including the Allegheny Health Network (AHN)'s Islet Cell Isolation Laboratory. The AHN group led by Massimo Trucco and Rita Bottino has a background in clinical islet isolation as part of the clinical total pancreatectomy and autologous islet transplantation procedure (47). The observation that each pancreas is unique in properties that influence islet isolation and the experience borne of the necessity to optimize islet yield for each patient teaches that flexibility within established parameters is needed to maximize the benefits of islet transplantation. Free from the strict isolation protocols in which islet allotransplantation teams are trained, islet autotransplantation teams develop flexible case-by-case protocols to isolate islets from diseased organs. AHN has used this experience to successfully isolate islets from marginal donors including pediatric, geriatric, obese, and those diagnosed with autoimmune and other diseases (data not published). This research demonstrates the ability to rescue islets from organs that would not be selected to provide islets under standard islet allotransplantation protocols. While we hope that one-day, islets from marginal donors, particularly donors that do not meet size or age requirements/recommendations, could provide a clinical recourse for patients with T1D, we continue to learn valuable lessons that can help to improve islet yields across the board. The addition of controlled flexibility into CIT-07-based protocols may help to overcome the failure to produce transplantable lots by not achieving sufficient islet mass to proceed without impacting product identity, efficaciousness, and safety.

While the CIT-07 clinical trials have demonstrated that islet allotransplantation has matured as a therapy and can produce lasting and appreciable improvements in the QOL of patients with T1D and SHEs, investigators seeking additional improvements have several areas in which to work.

Islet transplant location is one area of inquiry that was thought settled decades ago but has now been reopened as investigators seek a more optimal site. As long ago as the early 1970s, the liver via infusion into the portal vein was broadly accepted as the best location to transplant islets in rodents. Due to the prevalence and importance of rodent research, this principle became generalized to most animal models as the field expanded and became, almost exclusively, the clinical site of preference. Additional research has since determined that the liver site was less than optimal for several reasons including, (1): contact with blood stream leading to IBMIR thus reducing islet mass by up to 50%, (2): potential for thrombosis during infusion, (3): relatively low oxygen tension compared to the pancreas (61, 62). Investigators have looked to the kidney capsule, omental pouch, gastric submucosa, peritoneal space, spleen, bone, and muscle among other potential sites. Animal models have identified particular advantages in several alternative sites, such as low blood contact to reduce IBMIR, or the ability to biopsy the site after islet delivery, however so far these positives are canceled with equally compelling negatives such as poor oxygen supply, difficulty of surgical procedure, or need for more islets to correct glycemic imbalance. Ongoing research may eventually produce a better site for islet infusion, however, despite its now recognized limitations, the liver remains the best choice at present for clinical islet allotransplantation. Table 1 reports a list of sites used in clinical islet transplantation.

**Table 1:** A list of sites used for islet transplantation in humans with main features affecting clinical utility.

**Site**	**Pros**	**Cons**
Liver (31)	Well characterized in humans Clinically effective Safe, minimally invasive Physiologic release of insulin Blood exposure	Highly active immune system Early islet loss due to IBMIR and hypoxic apoptosis Risk of portal hypertension
Spleen (22)	Reduced risk of portal hypertension Physiologic release of insulin Blood exposure	Limited clinical experience Early islet loss due to IBMIR
Renal capsule (63)	Well-established rodent model Potentially less IBMIR Potential for biopsy	Tight capsule in larger animals Poor initial clinical results Systemic release of insulin
Omental pouch (64)	Well-established small and large animal models Potentially less IBMIR Physiologic release of insulin Potential for biopsy	Not well characterized in humans More invasive than other options Requires higher # of islets to restore glycemic control
Skin (65)	Good safety profile Easy to implant, biopsy, and re-transplant if necessary	Poor vascularization Systemic release of insulin
Muscle (66)	Rich vascularization Potentially less IBMIR Minimally invasive Potential for biopsy	Systemic release of insulin
Bone marrow (67)	Protected microenvironment Potentially less IBMIR Easy to implant	Limited clinical experience Systemic release of insulin

Further on the frontiers of medicine are alternative methods to produce islets without relying on human organ donors, including techniques for the xenotransplantation of porcine islets. Greek myths of creatures that were part human and part animal such as the centaur or minotaur show man's early fascination with the mingling of human and animal characteristics. CG Groth reported the first xenotransplantation of porcine fetal islet-like cell clusters in patients with T1D in 1994. While this study demonstrated the feasibility of porcine islet transplantation it did not show improvement in patients (68). In the subsequent decades, porcine islet xenotransplantation has been more fully explored in pre-clinical trials using nonhuman primates (69, 70). Humoral rejection represents a major obstacle for successful xenotransplantation. Epitopes of α1,3Gal are found on the surface of almost all animals, with the significant exception of humans and some primates, and represents the primary antigen causing hyperacute rejection in pig to human xenotransplantation and pig to nonhuman primate islet xenotransplantation. A collaborative effort between the University of Pittsburgh and Revivicor, Inc. led to the generation of Gal1,3-galactosyltransferase gene-knockout (GTKO) pigs which do not express Gal (71). This proved to be a major milestone in the advancement of xenotransplantation. While additional xenoantigens have since been discovered, Gal remains the most relevant, and GTKO pigs are recognized as the likely background of choice for eventual clinical translation. The knock-out of Gal, however, did not prevent islet rejection and other gene manipulations were also explored. In 2009, the islet group in Pittsburgh was the first to demonstrate long-term islet graft function (for up to 1 year) in streptozotocin induced diabetic nonhuman primates transplanted with pig islets genetically modified to express a human complement-regulatory protein (hCD46). The hCD46 expressed on the pig islets limited antibody-mediated rejection, which, in turn, allowed for a reduction in immunosuppression needed to preserve sufficient islet mass to sustain long-term normoglycemia. However, it did not reduce initial islet loss associated with IBMIR as it was expected to do (72). This led to further development of multi-genetic pig islet donors that would be able to provide multi-faceted protection to enhance islet engraftment. Five years later, the same group achieved a similar success with the first long-term engraftment of islets from a multi-transgenic pig. A pig with 4 modified genes, (i), GTKO, and the expression of (ii), hCD46, (iii), human tissue factor pathway inhibitor (hTFPI) for anti-throbosis and anti-inflammatory effects, and (iv), CTL4-Ig to inhibit the cellular immune response, demonstrated improved success in preserving islet mass during the early post-transplant period and successfully maintained islet engraftment and function for up to 1 year (73). This study also provided some of the first insights into glucose metabolism in pigs expressing human genes regulated by an insulin promoter, demonstrating that multiple islet targeted transgenes inserted into pigs were not detrimental to islet function and opening the door for even further experimentation and genetic manipulation in islet xenotransplantation (74). Multi gene donor pigs have proven to be an efficacious islet source in pig-to-non human primates reproducibly (75). An ongoing study with major implications for islet xenotransplantation is being carried out by CG Park and colleagues at the Seoul National University in Korea, where they have successfully maintained normoglycemia in diabetic primates after pig islet transplantation for >600 days (76). One common thread between these successful long-term pig to nonhuman primate islet studies is their use of an anti-CD154 monoclonal antibody (mAb) based immunosuppression to prevent rejection. Despite evidence that anti-CD154mAb can be effective and safe in pig to nonhuman primate models of islet transplantation (77), it has been associated with thromboembolic complications in humans and is not clinically translatable. Although very promising data are emerging on the use of anti-CD40 antibodies (costimulation blockers) in organ xenotransplantation (78, 79), the islet xenotransplantation community is still searching for a clinically translatable immunosuppression that can successfully prevent rejection without excessive side effects. New technology for targeted genome editing, notably clustered regularly interspaced short palindromic repeats (CRISPR)-associated protein-9 nuclease (Cas9), offers hope that more genetic manipulations of pig islets may improve compatibility between host and donor to the point that rejection may be successfully controlled with the use of a previously untenable immunosuppression. The field of islet xenotransplantation is steadily advancing and may be approaching a critical mass of experience and technique to begin clinical trials soon (80).

Islet encapsulation is another advanced method of islet transplantation in which isolated islets of either human or pig origin can be transplanted without the need for toxic immunosuppression. This would prove to be especially beneficial for pig islet xenotransplantation. The encapsulation of islets with a semi-permeable barrier that allows the exchange of nutrients and hormones including insulin while maintaining immune-isolation would overcome one of the chief obstacles of xenotransplantation. While clinical trials of porcine islets have been carried out with some success in New Zealand and Argentina (81), more research to develop optimal encapsulation methods and materials may be necessary before the technique is ready for larger clinical trials in the US. Looking even slightly more to the future, although not yet practicable, the prospect of human stem cell derived islets may one day completely eliminate the need for organ donors to provide islets (82).

## The cost of islet transplantation

While support from the islet community for these cutting edge endeavors may not be universal, perhaps the one point that everyone can agree upon is that islet transplantation is expensive. In 2009, the cost of organ procurement for islet transplantation was $20,000, with an additional $20,000 for isolation costs and $5,000 in hospital costs. With a success rate of ~50%, single donor islet transplantation can easily cost more than $80,000 and costs for multiple islet infusions can top $120,000 (83). These figures represent the cost of the transplantation and do not include the immunosuppression that patients would be required to take in order to prevent rejection, which would be continued over the life of the patient. A comparative cost analysis of islet versus pancreas transplantation reported in 2016 placed the cost for islet transplantation at $138,872, substantially in line with previously reported data (54). Immunosuppression would be required for either pancreas or islet transplantation with similar costs for each.

At the moment, the islet community is poised on a precipice and we are still waiting to see who will take the next important step toward FDA licensure. When islet allotransplantation loses its experimental designation and becomes a fully integrated clinical treatment the bulk of expense will be covered by third party reimbursement, however, patients may still receive potentially large bills too, and many centers may take a wait and see approach to determine the financial feasibility of performing the clinical procedure. Centers will be cautious because the expense necessary to maintain Current Good Manufacturing Practices and Current Good Tissue Practices will be significant and even after third party reimbursement becomes standard, it may take time for demand to support the expenditures incurred in start-up. The irony of the situation is that while CIT-07 has shown the way for successful islet allotransplantation, the cost of the facilities, and costs associated with organ procurement and the procedure, make it unlikely that, at least at first, many centers would decide to offer the therapy. We may see reorganization of the field, where large regional centers, perhaps already affiliated with the CITC, dominate the clinical landscape. In this scenario, where only large regional centers are able to afford to provide clinical islet allotransplantation, smaller centers can shift their focus to research that may enhance or optimize the procedure or advance alternate islet sources through xenotransplantation, encapsulation or stem cell research.

The cost of organ procurement and the specialized skill and experience necessary to isolate islets, however, continues to present a challenge for research. The Integrated Islet Distribution Program (IIDP), established with support from the Juvenile Diabetes Research Foundation (JDRF) and NIH NIDDK in 2009, is the latest in a line of organizations that offer to provide islets to researchers across the US for a fee. While the idea is admirable, the implementation is still inadequate to meet many researchers needs. Some investigators already feel that access to the human islets is becoming more difficult to obtain, potentially limiting research (84). One potential solution to this “crisis” would be for the NIH or a group of diabetes research organizations including the JDRF or the American Diabetes Association (ADA), to fund a nonprofit entity that would match select individual researchers and islet isolation centers to provide islets suiting their needs, coordinate logistics, and provide fair reimbursement for services. This may provide an equitable method of distribution among researchers and encourage islet isolation centers to establish strong working relationships with outside researchers to provide more high quality islets for study and the flexibility to customize islets to demand. In essence the fully funded nonprofit would act as a giant grant mechanism to provide islets that would be shared among many eligible researchers instead of only funding an individual proposal. Funds could be allocated from organizational donations or additional government funding. NIH grants funding proposals for islet research can direct applicants to match with an appropriate isolation center. The AHN islet isolation center already acts in a similar capacity for a network of researchers across the US (85–88) acting under grants awarded to investigators who found that IIDP or similar organizations could not adequately meet their need for specialized islets.

Larger centers performing islet allotransplantation would be reimbursed for the procedure and some of this revenue would be available to advance future clinical trials.

To date, no group has been granted licensure with the FDA and it is unknown how many will pursue the Biologics Licence Application following CIT guidelines so it is unclear what the future holds for islet transplantation. However, there is no going back for islet transplantation and after the decades of study, trials, and resources invested to get this far, the islet community must push forward to financially legitimize a proven technology that provides another option to improve or cure diabetes.

## Author contributions

RB and MT chose the topic and contributed sections for the manuscript, MK wrote the first draft of the manuscript, RB, MT, MK, CK, and SB contributed to the revision of the manuscript. CK and SB provide images and appropriate legends. All authors read and approved the submitted version.

### Conflict of interest statement

The authors declare that the research was conducted in the absence of any commercial or financial relationships that could be construed as a potential conflict of interest.

## Abbreviations

ADA: American Diabetes Association
AHN: Allegheny Health Network
ATG: Antithymocyte globulin
BMI: Body mass index
CITC: Clinical Islet Transplant (CIT) Consortium
CITR: Collaborative Islet Transplant Registry
CRISPR-Cas9: Clustered regularly interspaced short palindromic repeats (CRISPR)-associated protein-9 nuclease (Cas9)
ECM: Extra cellular matrix
ESRD: End Stage Renal Disease
GTKO: Gal1,3-galactosyltransferase gene-knockout
hTFPI: Human tissue factor pathway inhibitor
HTK: Histidine-tryptofane-ketoglutarate
IAH: Impaired awareness of hypoglycemia
IEQ: Islet equivalent quotient
IIPD: Integrated Islet Distribution Program
JDRF: Juvenile Diabetes Research Foundation
mAb: Monoclonal antibody
NIAID: National Institute of Allergy and Infectious Diseases
NIDDK: National Institute of Diabetes and Digestive and Kidney Diseases
NIH: National Institutes of Health
NNCIT: Nordic Network for Clinical Islet Transplantation
PFC: Perfluorinated compound
PHPI: Purified human pancreatic islet
PVT: Portal vein thrombosis
QOL: Quality of Life
SHE: Severe hypoglycemic event
T1D: Type 1 diabetes
TNF: Tumor necrosis factor
UW: University of Wisconsin.
